# The illusory certainty: Information repetition and impressions of truth enhance subjective confidence in validity judgments independently of the factual truth

**DOI:** 10.1007/s00426-024-01956-7

**Published:** 2024-03-25

**Authors:** Annika Stump, Andreas Voss, Jan Rummel

**Affiliations:** Institute of Psychology, Heidelberg University, Hauptstrasse 47-51, D-69117 Heidelberg, Germany

**Keywords:** Processing fluency, Information repetition, Subjective confidence, Truth judgments, Retention interval length

## Abstract

People not only judge repeatedly perceived information as more likely being true (the so-called truth effect) they also tend to be more confident after judging the validity of repeated information. These phenomena are assumed to be caused by a higher subjective feeling of ease (i.e., fluency) when processing repeated (vs. new) information. Based on the suggestion that a higher number of coherent mental activations is promoting a fluency experience, we argue that besides repetition an already existing information network, that is (nonspecific) prior knowledge, can enhance fluency. Following this argumentation, information repetition as well as the act of judging incoming information as being true (vs. false) should feed into subjective confidence – independently of the factual truth (when judging under uncertainty). To test this, we reanalyzed two published data sets and conducted a new study. In total, participants (*N* = 247) gave 29,490 truth judgments and corresponding ratings of subjective confidence while attending two judgement phases (i.e., 10 min and 1 week after the exposure phase in each experiment). Results showed that (a) repetition (in 3 of 3 data sets) and (b) impressions of truth (in 2 of 3 data sets) were systematically related to higher subjective confidence. Moreover, we found (c) a significant positive interaction between repetition and impressions of truth after both intervals in all data sets. Our analyses further underline the moderating effect of time: Influences of repetition significantly decreased with increasing time interval. Notably, the factual truth did not systematically affect any of the above reported effects.

Information forms the basis for our judgments, opinions, and behavior. Sharing of information never seemed easier, and the resulting amount of available information greater, given the multiple channels of information that have emerged, especially via the internet. However, these developments do not only entail benefits. For example, the NATO has recently provided information about the so-called information warfare, highlighting the importance and timeliness of topics like disinformation and misinformation in the light of rapid technological developments (NATO, 2020). Given these current developments, it is important to gain a better understanding of the mechanisms shaping our beliefs. Therefore, it is crucial to investigate not only the mechanisms underlying our instantaneous validity judgments but also factors that can lead us to question our initial judgments, or to look for additional information, such as the subjective confidence of decision makers (see e.g., Desender et al., 2018). Our research offers novel empirical findings in this understudied context, while underlining how existing theorical approaches can be used to explain observed repetition-based phenomena not only with respect to truth judgments, but also regarding subjective confidence in judgments of truth.

A widely known phenomenon when it comes to judging information validity is the illusory truth effect: Information presented repeatedly is more likely to be judged as “true” in comparison to novel information (Hasher et al., 1977). The vast majority of researchers in the field assume that the illusionary truth effect is mainly based on perceived high processing fluency caused by the repetition. Different studies have demonstrated that the truth effect can be induced by conceptual fluency (see e.g., Arkes et al., 1991; Hawkins et al., 2001) as well as perceptual fluency manipulations (see e.g., Reber & Schwarz, 1999; Garcia-Marques et al., 2016). In fact, the relative processing fluency appears to be crucial for the emergence of a truth effect. Dechêne et al. (2009) found an illusionary truth effect only when disfluent and fluent stimuli were presented intermixed. In classic truth effect experiments, fluency is usually manipulated through the presentation of novel and repeated information (i.e., stimuli are already presented during a former phase of the experiment, often referred to as exposition phase). As Garcia-Marques et al. (2016) argue, due to the verbatim as well as semantic repetition, this manipulation form can be seen as a source of both perceptual and conceptual fluency (see also Garcia-Marques et al., 2015). Unkelbach and Rom (2017) recently proposed a theoretical account aiming to provide a more comprehensive theory for the repetition-based truth effect. The authors argue that corresponding references in memory assign meaning to the information presented, and that a relatively high number of corresponding references, which should be linked coherently, results in more fluent information processing. Unkelbach and Rom (2017) further argue that information repetition can increase the number of coherently linked references, leading to an experience of higher processing fluency and an illusory truth effect. Taken together, this theoretical approach offers a comprehensive explanation for the occurrence of a repetition-based fluency experience and takes memory-based processes into account.

Recent findings already demonstrated that the repeated presentation of information (considered as a source of perceptual and conceptual fluency) increases not only validity judgments (i.e., the truth effect) but also subjective confidence in judgments of truth (e.g., Stump et al., 2022). In the present study, we aimed to apply and extend the referential theoretical account to better understand cognitive processes underlying subjective confidence in validity judgments. We argue that not only repetition but also nonspecific prior knowledge (i.e., prior knowledge related to the information that has to be evaluated, but not sufficient for an accurate assessment) can provide more references in the information network, at least when linked coherently. Consider, for example, the statement: “The California Condor is the world’s largest bird of prey.” For this statement, there might exist coherent knowledge in memory, such as: California has large birds of prey, and Condors are indeed known as a large species of bird. Since this knowledge forms coherently linked references for the statement’s components, a high experience of processing fluency is expected, resulting in an increased probability of judging this piece of information as true and strengthening the subsequent subjective confidence in this judgment. Now, in contrast, consider the statement: “The California Sparrow Hawk is the world’s largest bird of prey.” Although all elements of the statement have similar corresponding references in memory (i.e., region name, name of bird, qualifier about size), they lack coherence. Sparrows and Hawks, unlike Condors, are not associated with large species of birds.

This line of argumentation can be harmoniously integrated into the recently published Conviction Narrative Theory (CNT, “A theory of choice under radical uncertainty”) by Johnson et al. (2023). The CNT proposes that individuals use narratives, which can be considered as structured higher-order mental representations, for making sense of data and subsequent decisions under uncertainty. We propose that the strength of these narratives increases with a greater number of coherently linked references, which goes hand in hand with increasing ease of processing, subjective truth, and confidence. Similarly, Kahneman (2011) commented over a decade ago: “The confidence that individuals have in their beliefs depends mostly on the quality of the story they can tell about what they see, even if they see little. We often fail to allow for the possibility that evidence that should be critical to our judgment is missing – what we see is all there is.” Kahneman (2011, p. 87).

Based on our theoretical argumentation, we assume that both repetition and the act of judging a statement as being true (vs. false) may increase subjective confidence. It is important to point out that previous research indicates certain contextual variables that can impact the repetition-based effects. For instance, researchers have observed effects of the experimental instructions (e.g., Jalbert et al., 2020), the way in which information is encoded (e.g., Brashier et al., 2020; Riesthuis & Woods, 2023), the processing depth during initial presentation (e.g., Unkelbach & Rom, 2017), the frequency of repetitions (e.g., Hassan & Barber, 2021), and the length of the retention interval (e.g., Henderson et al., 2021) on repetition-based illusory truth. Noteworthy, the length of the retention interval has already been observed to moderate the effect of repetition on judged truth as well as subjective confidence (Stump et al., 2022). As a result, we furthermore implemented two retention interval lengths (10 min/1 week) in our additional (third) study and expected to replicate the moderating effect of the retention interval length on both repetition-based effects.

In the following, we provide a short description of two data sets (Stump et al., 2022) that we reanalyzed in the present paper. The data sets are publicly available from: https://heidata.uniheidelberg.de/dataset.xhtml?persistentId=doi:10.11588/data/E3JAHX. Furthermore, we analyzed data from an additional (larger) study which was designed to address several research questions. In the following, we describe the procedures and measures which are relevant to the present hypotheses. The new data is publicly accessible at https://heidata.uni-heidelberg.de/dataset.xhtml?persistentId=doi:10.11588/data/WDQJWW.

## The present research

All analyzed data sets are based on an experimental design including five phases. During the first phase of each experiment, the *exposure phase*, statements from two statement sets were displayed trial-wise in random order, and participants had to classify each statement into one of six semantic categories (e.g., geography, biology). The second experimental phase was a *short retention interval* (10 min), during which participants worked on a nonverbal filler task. Afterwards, the *first judgment phase* (third experimental phase) started, in which 30 repeated (i.e., statements already presented during the exposure phase) and 30 novel statements were displayed trial-by-trial in random order. For each statement participants judged the truth (“true” vs. “false”) and subsequently rated the confidence in their previously made truth judgment (1 “very uncertain” to 6 “very certain”). After a *longer retention interval* of 1 week (the fourth experimental phase) the *second judgment phase* (the fifth experimental phase) took place. The procedure in the second judgement phase was identical to the one of the first judgment phase, except that different statement material was used. That is, statements from the final unused stimulus set were taken and presented intermixed with statements already displayed in the exposition phase which had not been presented in the first judgment phase.

We reanalyzed two data sets from previous truth effect experiments, which include 60 truth judgments and corresponding ratings of subjective confidence per judgment phase (Stump et al., 2022) – in the following results section referred to as data set A (Exp. 1: *N* = 97) and data set B (Exp. 2: *N* = 75). The main aim of the published work (Stump et al., 2022) was to investigate potential cognitive and affective mechanisms underlying the repetition-induced truth effect. For this purpose, manipulations of (a) short-term affective states using subliminal presentations of emotional faces prior to each truth judgment (Exp. 1) or (b) the presence of an irrelevant source for potential changes in affective states (Exp. 2) have been included in the basic experimental design described above. A detailed description of the methods can be found in the original publication. Stump et al. (2022) also published additional analyses of subjective confidence ratings in the appendix (A-C). These results reveal significant effects of information repetition on subjective confidence. Effects of the provided truth judgments on confidence ratings were not tested. Neither the manipulation of short-term affective states (Exp. 1) nor the presence of an irrelevant source for potential changes in affective states (Exp. 2) significantly influenced the subjective confidence.

For the present paper, we included data from an additional larger study, which was designed to address several research questions – in the following results section referred to as data set C (*N* = 75). In this study, no further experimental manipulations were included in the basic truth effect design described above. In contrast to the experiments reported above (Stump et al., 2022), the statements were not presented for a fixed time (3500 ms) but remained on the screen until the participants made their truth judgments (“true” vs. “false”) per key press. As noted, this larger study was planned to address several research questions, which is why fEMG activities were recorded during the experiment. The data set includes 60 truth judgments and corresponding ratings of subjective confidence per each judgment phase (*N* = 75).^1^ A detailed description of the methods can be found in the following section.

### Method – additional experiment (data set C)

#### Participants and design

A total of 81 participants were recruited from Heidelberg University using the recruitment software hRoot (Bock et al., 2014). Data from three individuals who did not attend the second experimental session, two individuals who aborted the experiment, and one participant who experienced a technical error were excluded. The remaining sample consisted of 75 participants, aged between 18 and 31 years (*M* = 21.63, *SD* = 2.96). Among them, 81% female individuals (1 person indicated diverse and 13 participants indicated male as their gender). The majority of participants (68%) were non-psychology students. Participants received course credit or a compensation of 15 Euros (approximately 16 US$) for their participation.

The study design included two within-subject factors: the repetition status of presented statements (new vs. repeated) and the retention interval (10 min vs. 1 week after the exposure phase).

#### Material

The main statement material comprised 120 statements in German language, organized into four statement sets. Since individuals tend to use subjective experiences for shaping their judgments especially when they have to decide under uncertainty, we selected our statements from material that had been carefully pretested to ensure that the items were challenging enough for most individuals (Nadarevic, 2010). Exemplary statements are “Bob Dylan works part-time as a winemaker.” or “Spanish is the official language in Angola.” (note that these are English translations of the original German statements). All statements had a similar length to ensure a comparable processing time. Likewise, only affectively neutral statements were chosen, as the statement’s content should not elicit any affective reactions. Each of the four sets consisted of 15 true and 15 false statements, with similar mean truth ratings and standard deviations across the sets (set A: *M* = 4.06, *SD* = 1.20; set B: *M* = 4.06, *SD* = 1.18; set C: *M* = 4.05, *SD* = 1.18; set D: *M* = 4.05, *SD* = 1.20). During both judgment phases, two sets of statements were used: one with new statements and one with statements already presented in the exposure phase. The assignment of different statement sets to the experimental phases has been counterbalanced across subjects.

#### Procedure

After obtaining consent and placing the electrodes on the participants’ skin, the computer experiment started. In the first experimental phase, statements from two sets were presented trial-by-trial, and participants had to categorize them into one of six predefined knowledge categories by pressing the corresponding number keys. To mitigate potential primacy and recency effects, six buffer statements were presented at the beginning and end of this phase. The 60 statements in between were taken from two statement sets (including 15 true and 15 false items, respectively) and presented in random order. Each trial began with a fixation cross displayed for 1 s, followed by a statement presented in the center of the screen until participants responded via key press. As for the following tasks, there was no time limit given per trial. However, subjects were instructed to make their responses as fast as possible while trying to avoid unnecessary mistakes.

After a subsequent 10-minute retention interval, during which participants engaged in a non-verbal filler task, the first judgment phase of the experiment began. In this phase, 30 new statements and 30 repeated statements were presented in random order, trial-by-trial. The 30 new statements were selected from one of the previously unused sets, while the 30 repeated statements came from one of the sets presented during the exposure phase. A fixation cross was presented for 1000 ms prior to each statement which was displayed until participants made their truth judgments by pressing the “W” key (German for true, “wahr”) or the “F” key (German for false, “falsch”). Subsequently, subjects rated their confidence in the truth judgment made on a scale of 1 (“very uncertain”) to 6 (“very certain”) using the number keys.

The second judgment phase took place exactly 7 days after the first experimental session. The procedure mirrored that of the first judgment phase, with the exception of using different material. That is, statements have been taken from the final unused set and presented intermixed with items from the exposition phase that were not shown during the first judgment phase a week earlier.

## Results

To account for the hierarchical data structure, a multilevel modeling approach was utilized for all analyses.^2^ Statistical analyses were performed with the software R (version 4.3.1) using the lme4 package by Bates et al. (2015; version 1.1–33) combined with the lmerTest package by Kuznetsova et al. (2017; version 3.1-3) to calculate *p*-values. A linear mixed model fit (maximum likelihood) was used. All models included random intercepts for subjects and statements.

The predictors (a) *truth judgment*, (b) *repetition status*, and (c) *factual truth* were dummy coded, using (a) “false” responses, (b) new statements, and (c) factual false statements as reference conditions.

### Response times

Since response times can serve as an indicator of processing fluency, we analyzed whether the predictors truth judgment and repetition status, which we theoretically have argued to be associated with increased processing fluency, can predict the response latencies.^3^ For this purpose, the predictors truth judgment, repetition status, judgment phase (coded − 0.5 for the first and + 0.5 for the second judgment phase) and the interactions including these variables were entered into the analysis. Results demonstrated significant negative main effects for repetition status (*b* = − 0.034, *p* = .002), truth judgment (*b* = − 0.035, *p* < .001), and judgment phase (*b* = − 0.053, *p* < .001), as well as significant interactions involving truth judgment and repetition status (*b* = − 0.093, *p* < .001) and truth judgment, repetition status and judgment phase (*b* = 0.124, *p* < .001). The findings indicate that participants made their truth judgments faster when a statement was repeated and they perceived a statement as true. The three-way interaction suggests that the time between first exposure and the later judgment phases significantly influenced the interaction effect of repetition status and truth judgment.^4^

To consider effects of the potential confounding variable *factual truth*, in addition to the predictors of the first model, the variable factual truth as well as all interactions involving the predictors repetition status, truth judgment, and factual truth were included in the model to predict the reaction times per trial. In addition to the findings reported above, no significant effects were observed, indicating that the reported findings are not affected by the factual truth of the presented statements.

To account for the influence of interval length and for the sake of a better comparability with other research (the majority of studies include only one interval length), all subsequent analyses were conducted for the first and second judgment phase separately.

### Subjective confidence (first judgment phase)

In a first model, the level-1 predictor *repetition status* was included to predict the subjective confidence in validity judgments made (see Table 1). The results reveal a positive main effect for repetition status in all data sets (data set A: *b* = 0.744, *p* < .001; data set B: *b* = 0.745, *p* < .001; data set C: *b* = 0.617, *p* < .001), implying significantly increased subjective confidence when judging the truth of repeated compared to new statements.

**Table 1:** Multilevel modelling results for the prediction of subjective confidence (Model 1)

			Fixed Effects					
		10-minutes interval			1-week interval			
*Data Set A*								
	*b*	*SE*	*t*	*p*	*b*	*SE*	*t*	*p*
Intercept	3.028	0.095	31.990	< 0.001***	2.900	0.095	30.555	< 0.001***
Repetition Status	0.744	0.035	21.250	< 0.001***	0.266	0.033	8.123	< 0.001***
*Data Set B*								
	*b*	*SE*	*t*	*p*	*b*	*SE*	*t*	*p*
Intercept	2.878	0.097	29.580	< 0.001***	2.717	0.098	27.633	< 0.001***
Repetition Status	0.745	0.043	17.140	< 0.001***	0.302	0.040	7.544	< 0.001***
*Data Set C*								
	*b*	*SE*	*t*	*p*	*b*	*SE*	*t*	*p*
Intercept	2.662	0.098	27.090	< 0.001***	2.505	0.099	25.205	< 0.001***
Repetition Status	0.617	0.040	15.540	< 0.001***	0.154	0.035	4.382	< 0.001***

*Notes. N* = 97 (Set A); *N* = 75 (Set B); *N* = 75 (Set C). ***p < .001

**Fig. 1:**
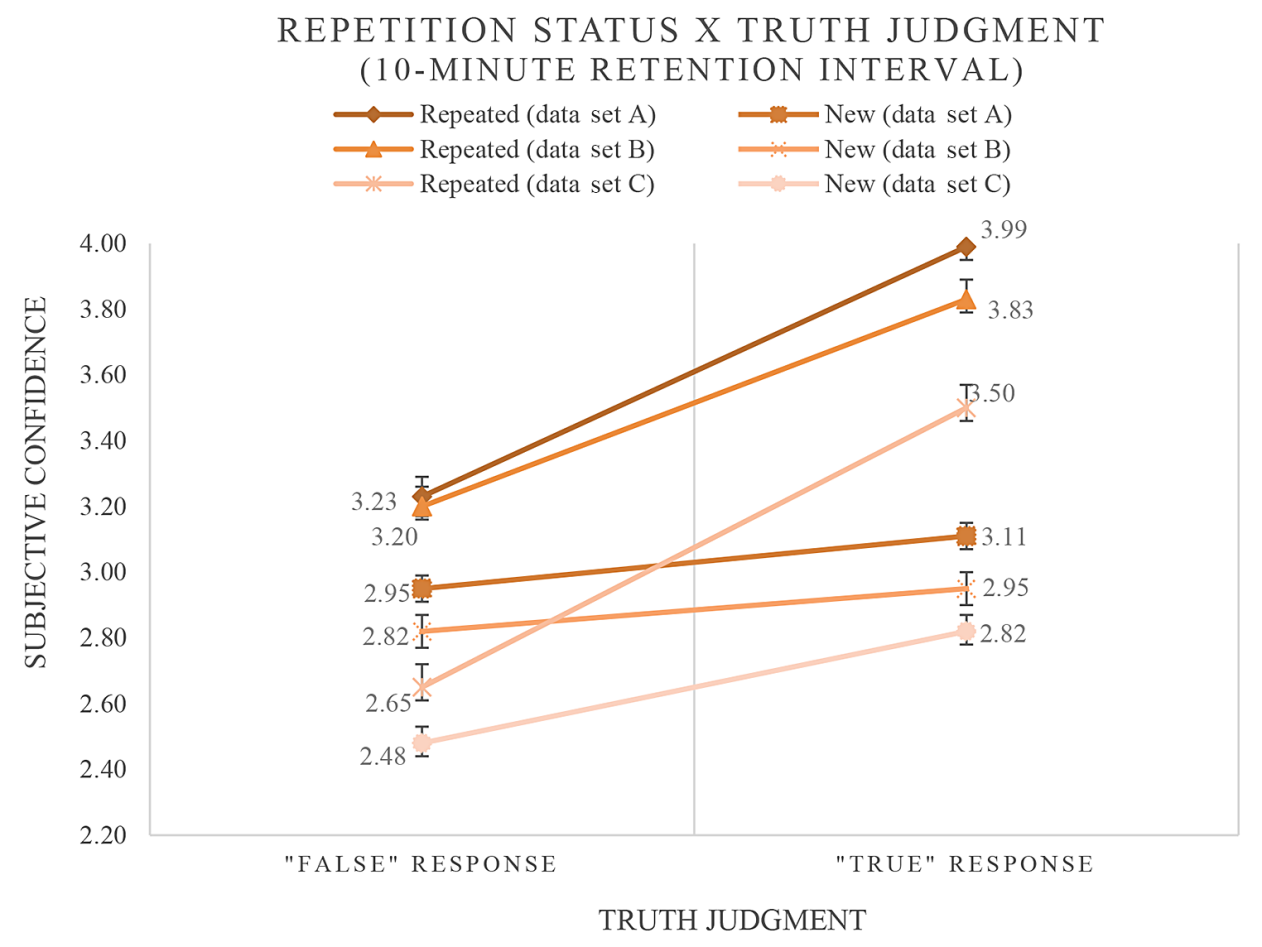
Mean ratings of subjective confidence for new and repeated statements as a function of truth judgments made (first judgment phase). *Note.* Mean ratings of subjective confidence for new and repeated statements were represented as a function of truth judgments made during the first judgment phase (10-minute retention interval)

In a next step, the predictors *repetition status*, *truth judgment* as well as an *interaction* involving both predictors were included in a second model (see Table 2). In addition to the main effect of repetition status (data set A: *b* = 0.408, *p* < .001; data set B: *b* = 0.413, *p* < .001; data set C: *b* = 0.229, *p* < .001), a main effect for truth judgment in two data sets (data set A: *b* = 0.257, *p* < .001; data set B: *b* = 0.038, *p* = .538; data set C: *b* = 0.290, *p* < .001) and a significant interaction in all data sets (data set A: *b* = 0.395, *p* < .001; data set B: *b* = 0.473, *p* < .001; data set C: *b* = 0.445, *p* < .001) were observed. The results imply that (a) repetition increased the subjective confidence even when the information was judged as being false (in all data sets), (b) increased subjective confidence after novel statements were judged as “true” (in data sets A and C), and (c) the validity judgments moderated the effect of information repetition on subjective confidence (in all data sets). The positive regression weights of the reported interaction effects indicate that in all data sets the repetition effects were markedly increased when participants perceived the respective information as true. Figure 1 illustrates the observed frequencies underlying these findings.

**Table 2:** Multilevel modelling results for the prediction of subjective confidence (Model 2)

			Fixed Effects					
		10-minutes interval			1-week interval			
*Data Set A*								
	*b*	*SE*	*t*	*p*	*b*	*SE*	*t*	*p*
Intercept	2.898	0.097	29.756	< 0.001***	2.809	0.098	28.590	< 0.001***
Repetition Status	0.408	0.059	6.960	< 0.001***	0.058	0.052	1.112	0.266
Truth Judgment	0.257	0.051	5.050	< 0.001***	0.166	0.048	3.461	< 0.001***
R. S. x T. J.	0.395	0.075	5.254	< 0.001***	0.300	0.068	4.429	< 0.001***
*Data Set B*								
	*b*	*SE*	*t*	*p*	*b*	*SE*	*t*	*p*
Intercept	2.859	0.101	28.397	< 0.001***	2.682	0.103	25.965	< 0.001***
Repetition Status	0.413	0.070	5.874	< 0.001***	0.083	0.064	1.295	0.195
Truth Judgment	0.038	0.062	0.615	0.538	0.063	0.059	1.067	0.286
R. S. x T. J.	0.473	0.091	5.195	< 0.001***	0.328	0.083	3.947	< 0.001***
*Data Set C*								
	*b*	*SE*	*t*	*p*	*b*	*SE*	*t*	*p*
Intercept	2.507	0.101	24.745	< 0.001***	2.386	0.103	23.128	< 0.001***
Repetition Status	0.229	0.069	3.331	< 0.001***	0.014	0.056	0.252	0.801
Truth Judgment	0.290	0.058	5.017	< 0.001***	0.212	0.052	4.094	< 0.001***
R. S. x T. J.	0.445	0.086	5.159	< 0.001***	0.192	0.073	2.632	0.009**

*Notes. N* = 97 (Set A); *N* = 75 (Set B); *N* = 75 (Set C). ***p < .001; **p < .01

To consider effects of the potential confounding variable *factual truth*, the predictors repetition status, truth judgment, factual truth as well as all interactions involving these variables were included to predict the subjective confidence in a following third model. Besides the findings already reported above, results show only one additional significant effect in data set B (truth judgment x factual truth, *b* = 0.344, *p* = .006), indicating higher confidence ratings after judging factual true statements as being true. However, the effects reported above were unaffected in data set B, and we did not find any influence of factual truth in the other two data sets, suggesting that impressions of truth as well as information repetition were systematically associated with increased subjective confidence independently of the factual truth of the presented statements.

Decreases in the Akaike information criterion (AIC) and Bayesian information criterion (BIC) indicated an improved model fit for the second model including the predictors repetition status, truth judgment and an interaction between both predictors (Set A: AIC = 20217.9; BIC = 20264.6; Set B: AIC = 16321.0; BIC = 16365.9; Set C: AIC = 15254.2; BIC = 15298.9) compared to the more parsimonious first model which included only repetition status as predictor (Set A: AIC = 20366.6; BIC = 20399.9; Set B: AIC = 16371.5; BIC = 16403.6; Set C: AIC = 15395.5; BIC = 15427.5), suggesting that, besides repetition, the individual impressions of truth and the interaction involving these predictors explained substantial variance in subjective confidence.

### Subjective confidence (second judgment phase)

As for the analyses of the confidence ratings in the first experimental session, the level-1 predictor *repetition status* was included in a first model to predict the subjective confidence in validity judgments made (see Table 1). A main effect of repetition status was found in all data sets (data set A: *b* = 0.266, *p* < .001; data set B: *b* = 0.302, *p* < .001; data set C: *b* = 0.154, *p* < .001), indicating that, also after the 1-week interval, information repetition resulted in higher subjective confidence in truth judgments made.

**Fig. 2:**
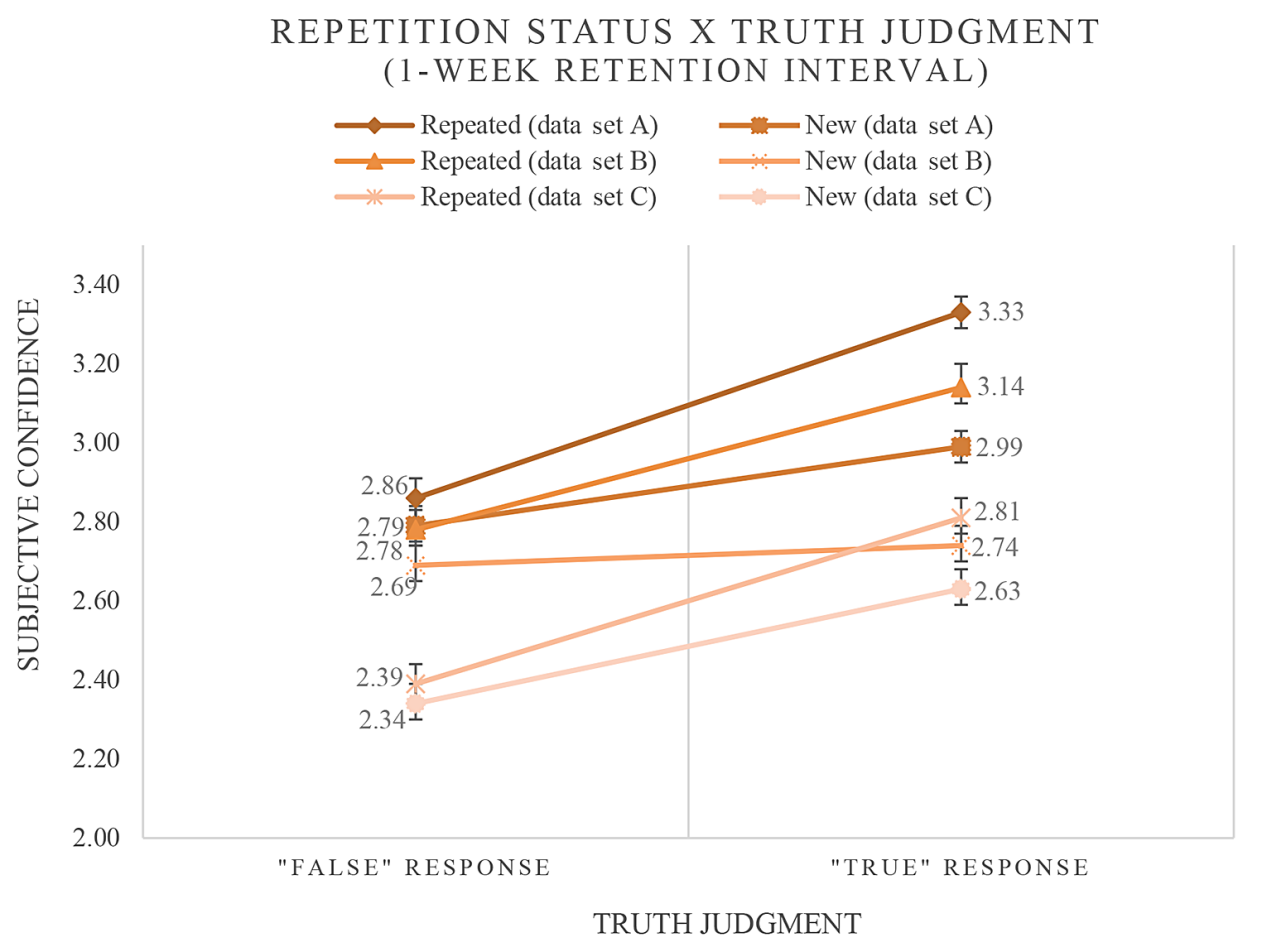
Mean ratings of subjective confidence for new and repeated statements as a function of truth judgments made (second judgment phase). *Note.* Mean ratings of subjective confidence for new and repeated statements were represented as a function of truth judgments made during the second judgment phase (1-week retention interval)

In a following second model, the predictors *repetition status*, *truth judgment* as well as an *interaction* involving both predictors were included (see Table 2). We found no significant main effect of repetition status in all data sets (data set A: *b* = 0.058, *p* = .266; data set B: *b* = 0.083, *p* = .195; data set C: *b* = 0.014, *p* = .801). Results reveal a significant main effect of truth judgment in two of three data sets (data set A: *b* = 0.166, *p* < .001; data set B: *b* = 0.063, *p* = .286; data set C: *b* = 0.212, *p* < .001) and again a significant interaction in all three data sets (data set A: *b* = 0.300, *p* < .001; data set B: *b* = 0.328, *p* < .001; data set C: *b* = 0.192, *p* = .009). The results imply that (a) after the longer (1-week) interval repetition did not increase the subjective confidence when the information was judged as false (in all data sets). However, (b) subjective confidence was increased when novel statements were judged as “true” (in data sets A and C). Again, (c) impressions of truth significantly increased the effects of information repetition on subjective confidence, as indicated by the positive regression weights of the interaction effects found in all data sets. Figure 2 illustrates the observed frequencies underlying these findings.

To consider effects of the possible confounding variable *factual truth*, the predictors repetition status, truth judgment, factual truth as well as all interactions involving these variables were included in a third model to predict the subjective confidence in judgments. In addition to the findings reported above, results show no significant effects, indicating that the reported findings were not affected by the factual truth of the presented statements.

Decreases in the Akaike information criterion (AIC) and Bayesian information criterion (BIC) indicated an improved model fit for the second model including the predictors repetition status, truth judgment and an interaction between both predictors (Set A: AIC = 19560.7; BIC = 19607.4; Set B: AIC = 15808.1; BIC = 15852.9; Set C: AIC = 14374.7; BIC = 14419.5) compared to the more parsimonious first model which included only repetition status as predictor (Set A: AIC = 19654.0; BIC = 19687.3; Set B: AIC = 15844.3; BIC = 15876.4; Set C: AIC = 14441.2; BIC = 14473.2), suggesting that, besides repetition, the individual impressions of truth and the interaction involving these and information repetition explained substantial variance in subjective confidence also in the second experimental session.

## Discussion

The central aim of the present research was to extend the understanding of mechanisms influencing the subjective confidence in judgments of truth. Based on the theoretical assumption that a higher amount of coherent mental activations during information processing promotes a fluency experience, we argue that both repetition as well as the already existing information network can increase the experienced processing fluency and subsequent validity judgments. Following this idea, repetition of information as well as the subjective impression of truth are expected to enhance the confidence in judgments made – independently of the factual truth of the information. For this purpose, we (re-)analyzed the data of two published and one novel truth effect experiments. The data of all experiments are composed of 247 subjects, each participant performed two judgment phases (10 min and 1 week after first exposure), resulting in 29,490 validity judgments and corresponding ratings of subjective confidence.

We observed a unique effect of information repetition (i.e., an effect of repetition in the case of “false” responses) on subjective confidence after the short (10-minute) interval in all data sets. Furthermore, extended analyses showed that the factual truth did not impact this effect of information repetition (in 3 of 3 data sets). The results underline the influence of repetition on subjective confidence in made truth judgments independently from the given judgment as well as factual truth and holds important practical implications facing the prevalence of misinformation, disinformation, fake news or conspiracy theories. The present findings suggest that correcting false information through the presentation of contradictory statements may even strengthen the subjective confidence in prior beliefs. As an applied example, assuming that people who are convinced that COVID vaccines are inherently harmful are likely to judge statements that emphasize the benefits of COVID vaccines as false. Transferring the present results to this scenario, these individuals may not only be likely to judge information emphasizing the benefits of COVID vaccines to be false, but they may also become more confident in their judgment. As another example, imagine a group of people who firmly believe that human activity has no impact on climate change. When confronted with statements that emphasize the significant role of human activity in climate change, they might not only dismiss this information as false, but also find that their confidence in their original belief is strengthened. This implies that efforts to challenge certain environmental beliefs may paradoxically lead to people becoming more resolute. A possibly better information strategy might be to share information that have the potential to modify certain references in the information network. In our first example, information strategies could use references to vaccination in general or already established vaccines in our society. Future research may investigate the influences of these different ways of information presentations not only in the respect of truth judgments but also concerning the subjective confidence in judgments made. Given previous research findings indicating that higher confidence results in decreased information seeking (Desender et al., 2018), this repetition-confidence link holds the potential to profoundly impact beliefs in our societies.

Our results moreover reveal a unique effect of merely judging an information as being true (i.e., an effect of the response when statements were presented for the first time) on subjective confidence after both the short (10-minute) and long (1-week) interval in two of three data sets (data sets A & C) as well as a significant interaction involving the predictors information repetition and truth judgment after both retention interval lengths in all data sets. These findings highlight that, in addition to the influence of information repetition, the agreement vs. disagreement with the information should be considered when investigating processes underlying subjective confidence in judgments of truth. The results suggest that apart from repetition the already existing information network (i.e., vague prior knowledge) can enhance the experienced processing fluency, impressions of truth and subsequent the subjective confidence in given judgments. This explanation remains speculative but appears well in line with (a) Unkelbach and Rom’s (2017) referential theory as well as the conviction narrative theory (CNT) by Johnson et al. (2023), (b) our extended analyses demonstrating that the factual truth did not systematically impact the reported effects and (c) the analyses of response latencies showing that repetition, the response “true” (vs. “false”) as well as the interaction between both are systematically related to decreased reaction times that are indicative for increased processing fluency.

Our findings furthermore underline the importance of time with respect to effects of information repetition on subjective confidence in judgments. The main as well as interaction effects involving the predictor information repetition markedly decreased with rising retention interval length between statement presentations in all three data sets. These findings are well in line with previous results (Stump et al., 2022) which already demonstrated a simultaneous reduction of a repetition-induced (a) truth effect as well as (b) confidence effect in judgments made as a function of retention interval length.

For complete transparency, we finally want to point out that our collected data is from young German adults, all of them university students. Thus, the generalizability of the effects to other cultural contexts, other age groups, or to groups with different educational backgrounds cannot be assessed based on the present data. Nonetheless, we assume that the results reflect fundamental cognitive mechanisms which should not be limited to the specific group examined in our study. Future studies are needed to critically examine further potential confounding factors, such as the way information is encoded during the exposure phase or the frequency of repetitions.

## Conclusion

With the present research, we demonstrate that information repetition and individual impressions of truth are uniquely and jointly related to (a) decreased decision times and (b) increased subjective confidence in validity judgments made. Importantly, these findings were not systematically affected by the factual truth of the presented information. Our results further highlight the importance of time on effects of information repetition: The impact of repetition on subjective confidence significantly decreased with increasing retention interval between statement presentations. Taken together, the present findings suggest that information repetition as well as subjective impressions of truth are highly relevant in understanding the factors that shape confidence in truth judgments.

## Footnotes

1 Due to technical errors in some trials, these had to be excluded. This concerned 1.8% in the first judgment phase and 1.5% in the second judgment phase.

2 In multilevel modeling, the level-2 sample size is often most relevant for statistical power (Maas & Hox, 2005). Simulation studies by Maas and Hox (2005) indicate that, for multilevel analyses, parameter estimations are of sufficient accuracy with at least 50 level-2 units (see in addition, Paccagnella, 2011). With 97 (data set A) and 75 (data set B & C) level-2 units (participants) all experiments were well in line with these recommendations.

3 Because only data set C contains reaction times suitable for this analysis (response times were measured from the statements onset until participants’ responses by key press), data sets A & B have not been considered for this investigation.

4 To better understand this three-way interaction, we conducted follow-up reaction time analyses separately for the first and second judgment phase (J.P.). For this purpose, the predictors truth judgment, repetition status and the interactions including these variables were entered into the models. The results showed that the interaction effect involving truth judgment and repetition status was markedly reduced with increasing retention interval length (J.P. 1: *b* = − 0.134, *p* < .001; J.P. 2: *b* = − 0.019, *p* = .295).
